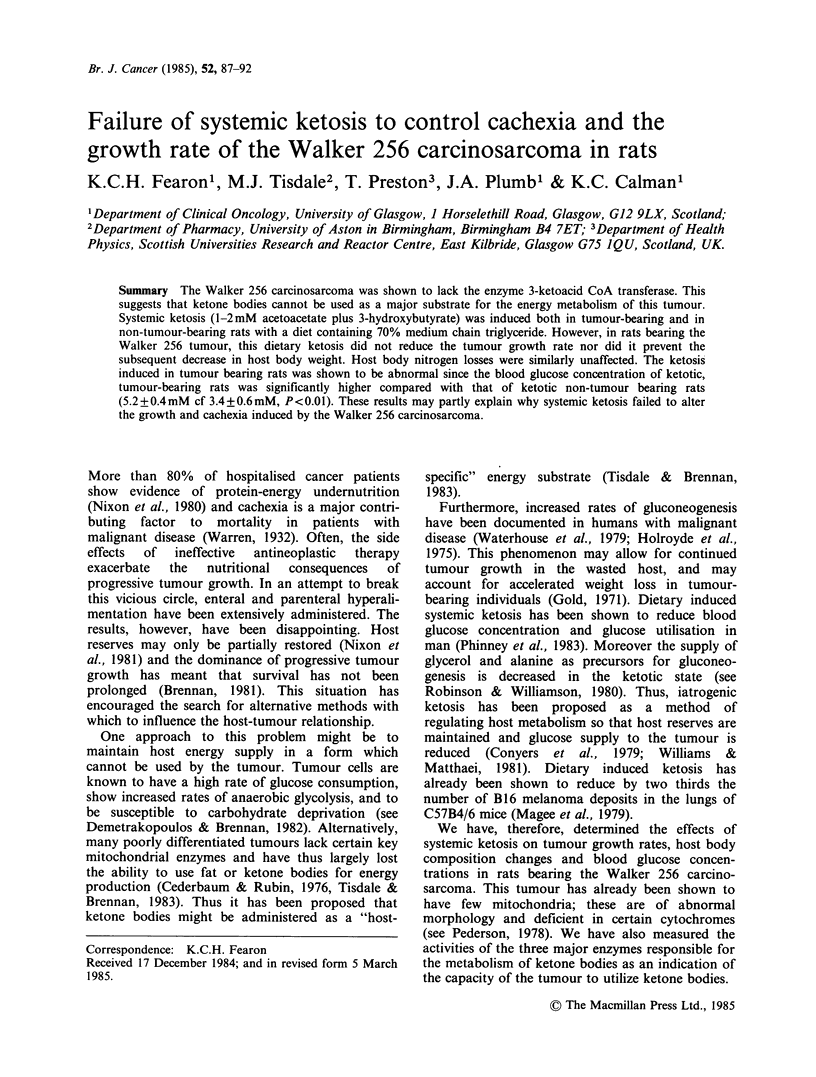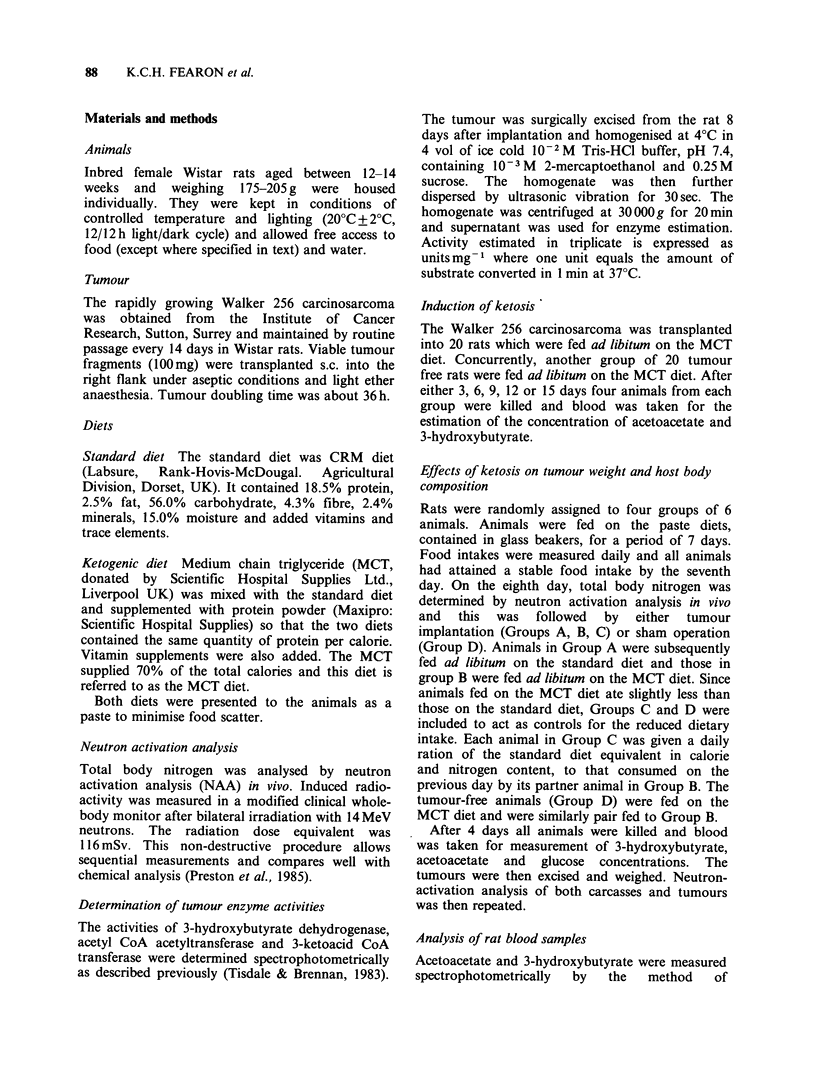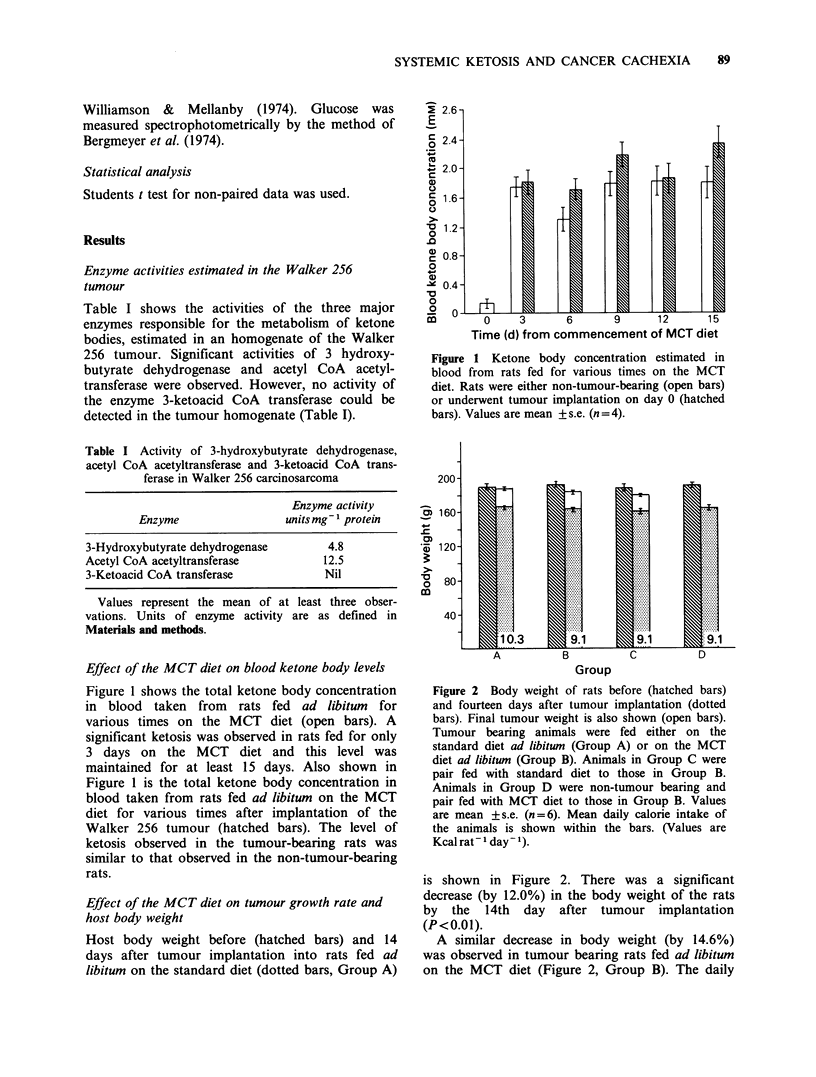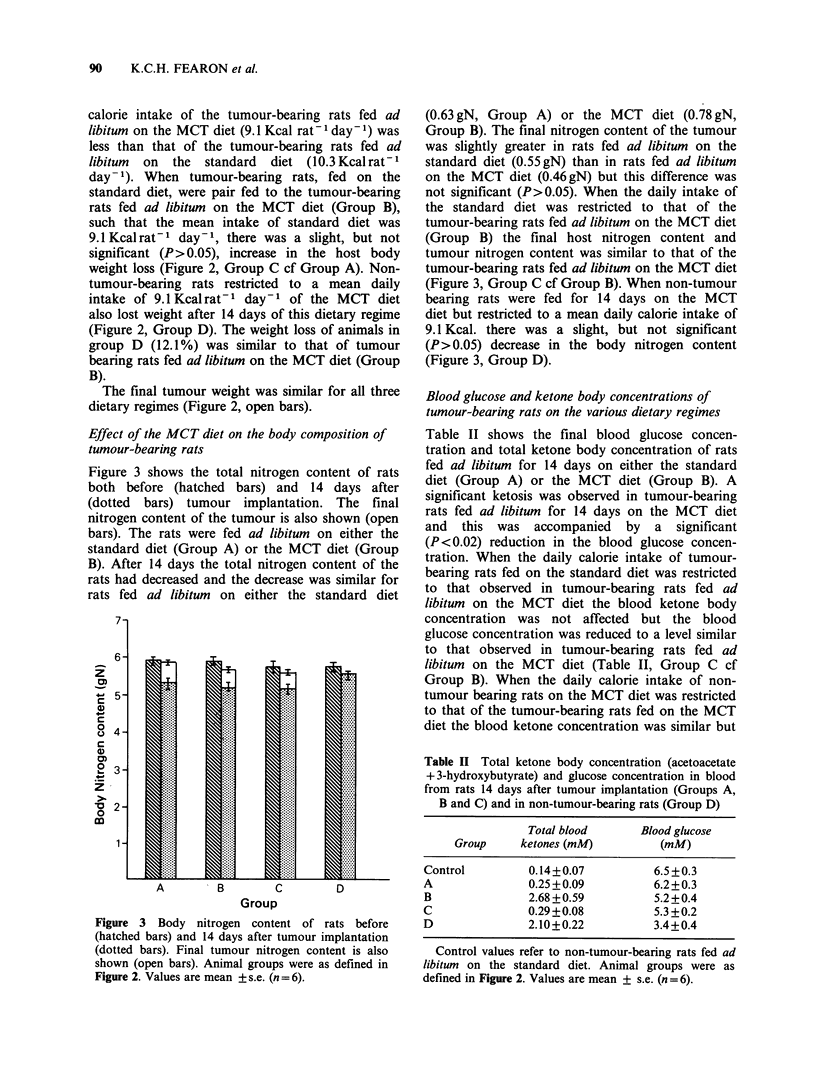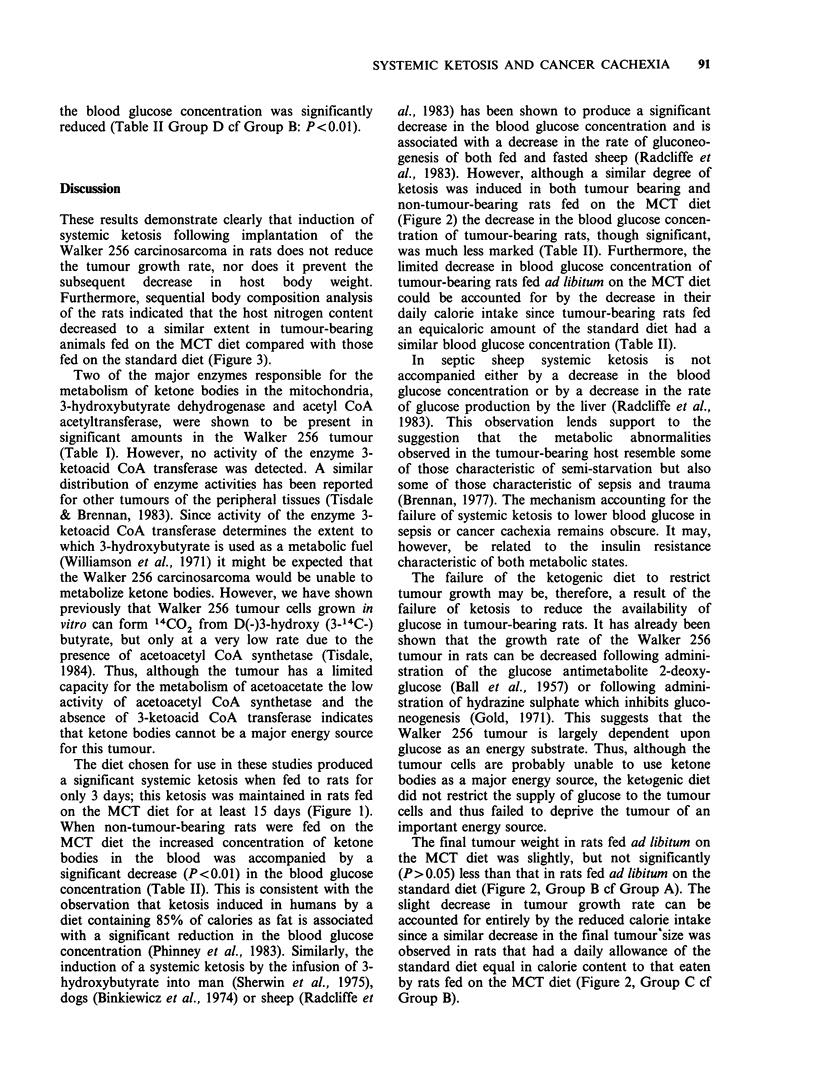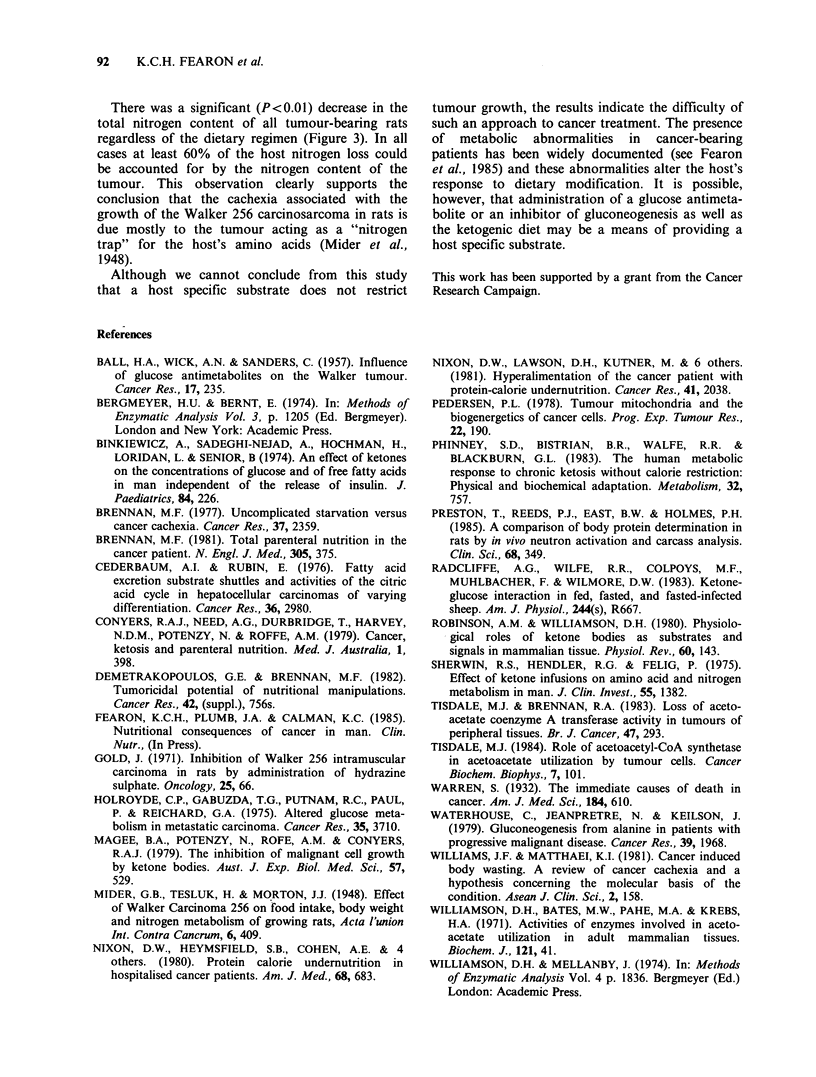# Failure of systemic ketosis to control cachexia and the growth rate of the Walker 256 carcinosarcoma in rats.

**DOI:** 10.1038/bjc.1985.153

**Published:** 1985-07

**Authors:** K. C. Fearon, M. J. Tisdale, T. Preston, J. A. Plumb, K. C. Calman

## Abstract

The Walker 256 carcinosarcoma was shown to lack the enzyme 3-ketoacid CoA transferase. This suggests that ketone bodies cannot be used as a major substrate for the energy metabolism of this tumour. Systemic ketosis (1-2 mM acetoacetate plus 3-hydroxybutyrate) was induced both in tumour-bearing and in non-tumour-bearing rats with a diet containing 70% medium chain triglyceride. However, in rats bearing the Walker 256 tumour, this dietary ketosis did not reduce the tumour growth rate nor did it prevent the subsequent decrease in host body weight. Host body nitrogen losses were similarly unaffected. The ketosis induced in tumour bearing rats was shown to be abnormal since the blood glucose concentration of ketotic, tumour-bearing rats was significantly higher compared with that of ketotic non-tumour bearing rats (5.2 +/- 0.4 mM cf 3.4 +/- 0.6 mM, P less than 0.01). These results may partly explain why systemic ketosis failed to alter the growth and cachexia induced by the Walker 256 carcinosarcoma.


*Br. J. Cancer* (1985), **52**, 87–92

### Failure of systemic ketosis to control cachexia and the growth rate of the Walker 256 carcinosarcoma in rats

K.C.H. Fearon[1], M.J. Tisdale[2], T. Preston[3], J.A. Plumb[1] & K.C. Calman[1]

[1]*Department of Clinical Oncology, University of Glasgow, 1 Horselethill Road, Glasgow, G12 9LX, Scotland;* [2]*Department of Pharmacy, University of Aston in Birmingham, Birmingham B4 7ET;* [3]*Department of Health Physics, Scottish Universities Research and Reactor Centre, East Kilbride, Glasgow G75 1QU, Scotland, UK.*

**Summary** The Walker 256 carcinosarcoma was shown to lack the enzyme 3-ketoacid CoA transferase. This suggests that ketone bodies cannot be used as a major substrate for the energy metabolism of this tumour. Systemic ketosis (1–2 mM acetoacetate plus 3-hydroxybutyrate) was induced both in tumour-bearing and in non-tumour-bearing rats with a diet containing 70% medium chain triglyceride. However, in rats bearing the Walker 256 tumour, this dietary ketosis did not reduce the tumour growth rate nor did it prevent the subsequent decrease in host body weight. Host body nitrogen losses were similarly unaffected. The ketosis induced in tumour bearing rats was shown to be abnormal since the blood glucose concentration of ketotic, tumour-bearing rats was significantly higher compared with that of ketotic non-tumour bearing rats ($5.2 \pm 0.4$ mM cf $3.4 \pm 0.6$ mM, $P < 0.01$). These results may partly explain why systemic ketosis failed to alter the growth and cachexia induced by the Walker 256 carcinosarcoma.

More than 80% of hospitalised cancer patients show evidence of protein-energy undernutrition (Nixon *et al.*, 1980) and cachexia is a major contributing factor to mortality in patients with malignant disease (Warren, 1932). Often, the side effects of ineffective antineoplastic therapy exacerbate the nutritional consequences of progressive tumour growth. In an attempt to break this vicious circle, enteral and parenteral hyperalimentation have been extensively administered. The results, however, have been disappointing. Host reserves may only be partially restored (Nixon *et al.*, 1981) and the dominance of progressive tumour growth has meant that survival has not been prolonged (Brennan, 1981). This situation has encouraged the search for alternative methods with which to influence the host-tumour relationship.

One approach to this problem might be to maintain host energy supply in a form which cannot be used by the tumour. Tumour cells are known to have a high rate of glucose consumption, show increased rates of anaerobic glycolysis, and to be susceptible to carbohydrate deprivation (see Demetrakopoulos & Brennan, 1982). Alternatively, many poorly differentiated tumours lack certain key mitochondrial enzymes and have thus largely lost the ability to use fat or ketone bodies for energy production (Cederbaum & Rubin, 1976, Tisdale & Brennan, 1983). Thus it has been proposed that ketone bodies might be administered as a "host-specific" energy substrate (Tisdale & Brennan, 1983).

Furthermore, increased rates of gluconeogenesis have been documented in humans with malignant disease (Waterhouse *et al.*, 1979; Holroyde *et al.*, 1975). This phenomenon may allow for continued tumour growth in the wasted host, and may account for accelerated weight loss in tumour-bearing individuals (Gold, 1971). Dietary induced systemic ketosis has been shown to reduce blood glucose concentration and glucose utilisation in man (Phinney *et al.*, 1983). Moreover the supply of glycerol and alanine as precursors for gluconeogenesis is decreased in the ketotic state (see Robinson & Williamson, 1980). Thus, iatrogenic ketosis has been proposed as a method of regulating host metabolism so that host reserves are maintained and glucose supply to the tumour is reduced (Conyers *et al.*, 1979; Williams & Matthaei, 1981). Dietary induced ketosis has already been shown to reduce by two thirds the number of B16 melanoma deposits in the lungs of C57B4/6 mice (Magee *et al.*, 1979).

We have, therefore, determined the effects of systemic ketosis on tumour growth rates, host body composition changes and blood glucose concentrations in rats bearing the Walker 256 carcinosarcoma. This tumour has already been shown to have few mitochondria; these are of abnormal morphology and deficient in certain cytochromes (see Pederson, 1978). We have also measured the activities of the three major enzymes responsible for the metabolism of ketone bodies as an indication of the capacity of the tumour to utilize ketone bodies.

Correspondence: K.C.H. Fearon
Received 17 December 1984; and in revised form 5 March 1985.

© The Macmillan Press Ltd., 1985

### Materials and methods

### Animals

Inbred female Wistar rats aged between 12–14 weeks and weighing 175–205 g were housed individually. They were kept in conditions of controlled temperature and lighting (20°C ± 2°C, 12/12 h light/dark cycle) and allowed free access to food (except where specified in text) and water.

### Tumour

The rapidly growing Walker 256 carcinosarcoma was obtained from the Institute of Cancer Research, Sutton, Surrey and maintained by routine passage every 14 days in Wistar rats. Viable tumour fragments (100 mg) were transplanted s.c. into the right flank under aseptic conditions and light ether anaesthesia. Tumour doubling time was about 36 h.

### Diets

*Standard diet* The standard diet was CRM diet (Labsure, Rank-Hovis-McDougal. Agricultural Division, Dorset, UK). It contained 18.5% protein, 2.5% fat, 56.0% carbohydrate, 4.3% fibre, 2.4% minerals, 15.0% moisture and added vitamins and trace elements.

*Ketogenic diet* Medium chain triglyceride (MCT, donated by Scientific Hospital Supplies Ltd., Liverpool UK) was mixed with the standard diet and supplemented with protein powder (Maxipro: Scientific Hospital Supplies) so that the two diets contained the same quantity of protein per calorie. Vitamin supplements were also added. The MCT supplied 70% of the total calories and this diet is referred to as the MCT diet.

Both diets were presented to the animals as a paste to minimise food scatter.

### Neutron activation analysis

Total body nitrogen was analysed by neutron activation analysis (NAA) *in vivo*. Induced radioactivity was measured in a modified clinical whole-body monitor after bilateral irradiation with 14 MeV neutrons. The radiation dose equivalent was 116 mSv. This non-destructive procedure allows sequential measurements and compares well with chemical analysis (Preston *et al.*, 1985).

### Determination of tumour enzyme activities

The activities of 3-hydroxybutyrate dehydrogenase, acetyl CoA acetyltransferase and 3-ketoacid CoA transferase were determined spectrophotometrically as described previously (Tisdale & Brennan, 1983). The tumour was surgically excised from the rat 8 days after implantation and homogenised at 4°C in 4 vol of ice cold $10^{-2}$ M Tris-HCl buffer, pH 7.4, containing $10^{-3}$ M 2-mercaptoethanol and 0.25 M sucrose. The homogenate was then further dispersed by ultrasonic vibration for 30 sec. The homogenate was centrifuged at 30 000 *g* for 20 min and supernatant was used for enzyme estimation. Activity estimated in triplicate is expressed as units $mg^{-1}$ where one unit equals the amount of substrate converted in 1 min at 37°C.

### Induction of ketosis

The Walker 256 carcinosarcoma was transplanted into 20 rats which were fed *ad libitum* on the MCT diet. Concurrently, another group of 20 tumour free rats were fed *ad libitum* on the MCT diet. After either 3, 6, 9, 12 or 15 days four animals from each group were killed and blood was taken for the estimation of the concentration of acetoacetate and 3-hydroxybutyrate.

### Effects of ketosis on tumour weight and host body composition

Rats were randomly assigned to four groups of 6 animals. Animals were fed on the paste diets, contained in glass beakers, for a period of 7 days. Food intakes were measured daily and all animals had attained a stable food intake by the seventh day. On the eighth day, total body nitrogen was determined by neutron activation analysis *in vivo* and this was followed by either tumour implantation (Groups A, B, C) or sham operation (Group D). Animals in Group A were subsequently fed *ad libitum* on the standard diet and those in group B were fed *ad libitum* on the MCT diet. Since animals fed on the MCT diet ate slightly less than those on the standard diet, Groups C and D were included to act as controls for the reduced dietary intake. Each animal in Group C was given a daily ration of the standard diet equivalent in calorie and nitrogen content, to that consumed on the previous day by its partner animal in Group B. The tumour-free animals (Group D) were fed on the MCT diet and were similarly pair fed to Group B.

After 4 days all animals were killed and blood was taken for measurement of 3-hydroxybutyrate, acetoacetate and glucose concentrations. The tumours were then excised and weighed. Neutron-activation analysis of both carcasses and tumours was then repeated.

### Analysis of rat blood samples

Acetoacetate and 3-hydroxybutyrate were measured spectrophotometrically by the method of

Williamson & Mellanby (1974). Glucose was measured spectrophotometrically by the method of Bergmeyer *et al.* (1974).

*Statistical analysis*

Students *t* test for non-paired data was used.

### Results

*Enzyme activities estimated in the Walker 256 tumour*

Table I shows the activities of the three major enzymes responsible for the metabolism of ketone bodies, estimated in an homogenate of the Walker 256 tumour. Significant activities of 3 hydroxybutyrate dehydrogenase and acetyl CoA acetyltransferase were observed. However, no activity of the enzyme 3-ketoacid CoA transferase could be detected in the tumour homogenate (Table I).

**Table I** Activity of 3-hydroxybutyrate dehydrogenase, acetyl CoA acetyltransferase and 3-ketoacid CoA transferase in Walker 256 carcinosarcoma

| *Enzyme* | *Enzyme activity units mg$^{-1}$ protein* |
|---|---|
| 3-Hydroxybutyrate dehydrogenase | 4.8 |
| Acetyl CoA acetyltransferase | 12.5 |
| 3-Ketoacid CoA transferase | Nil |

Values represent the mean of at least three observations. Units of enzyme activity are as defined in **Materials and methods**.

*Effect of the MCT diet on blood ketone body levels*

Figure 1 shows the total ketone body concentration in blood taken from rats fed *ad libitum* for various times on the MCT diet (open bars). A significant ketosis was observed in rats fed for only 3 days on the MCT diet and this level was maintained for at least 15 days. Also shown in Figure 1 is the total ketone body concentration in blood taken from rats fed *ad libitum* on the MCT diet for various times after implantation of the Walker 256 tumour (hatched bars). The level of ketosis observed in the tumour-bearing rats was similar to that observed in the non-tumour-bearing rats.

*Effect of the MCT diet on tumour growth rate and host body weight*

Host body weight before (hatched bars) and 14 days after tumour implantation into rats fed *ad libitum* on the standard diet (dotted bars, Group A)

**Figure 1** Ketone body concentration estimated in blood from rats fed for various times on the MCT diet. Rats were either non-tumour-bearing (open bars) or underwent tumour implantation on day 0 (hatched bars). Values are mean ±s.e. ($n=4$).

**Figure 2** Body weight of rats before (hatched bars) and fourteen days after tumour implantation (dotted bars). Final tumour weight is also shown (open bars). Tumour bearing animals were fed either on the standard diet *ad libitum* (Group A) or on the MCT diet *ad libitum* (Group B). Animals in Group C were pair fed with standard diet to those in Group B. Animals in Group D were non-tumour bearing and pair fed with MCT diet to those in Group B. Values are mean ±s.e. ($n=6$). Mean daily calorie intake of the animals is shown within the bars. (Values are Kcal rat$^{-1}$ day$^{-1}$).

is shown in Figure 2. There was a significant decrease (by 12.0%) in the body weight of the rats by the 14th day after tumour implantation ($P<0.01$).

A similar decrease in body weight (by 14.6%) was observed in tumour bearing rats fed *ad libitum* on the MCT diet (Figure 2, Group B). The daily calorie intake of the tumour-bearing rats fed *ad libitum* on the MCT diet (9.1 Kcal $rat^{-1} day^{-1}$) was less than that of the tumour-bearing rats fed *ad libitum* on the standard diet (10.3 Kcal $rat^{-1} day^{-1}$). When tumour-bearing rats, fed on the standard diet, were pair fed to the tumour-bearing rats fed *ad libitum* on the MCT diet (Group B), such that the mean intake of standard diet was 9.1 Kcal $rat^{-1}$ $day^{-1}$, there was a slight, but not significant ($P>0.05$), increase in the host body weight loss (Figure 2, Group C cf Group A). Non-tumour-bearing rats restricted to a mean daily intake of 9.1 Kcal $rat^{-1}$ $day^{-1}$ of the MCT diet also lost weight after 14 days of this dietary regime (Figure 2, Group D). The weight loss of animals in group D (12.1%) was similar to that of tumour bearing rats fed *ad libitum* on the MCT diet (Group B).

The final tumour weight was similar for all three dietary regimes (Figure 2, open bars).

*Effect of the MCT diet on the body composition of tumour-bearing rats*

Figure 3 shows the total nitrogen content of rats both before (hatched bars) and 14 days after (dotted bars) tumour implantation. The final nitrogen content of the tumour is also shown (open bars). The rats were fed *ad libitum* on either the standard diet (Group A) or the MCT diet (Group B). After 14 days the total nitrogen content of the rats had decreased and the decrease was similar for rats fed *ad libitum* on either the standard diet (0.63 gN, Group A) or the MCT diet (0.78 gN, Group B). The final nitrogen content of the tumour was slightly greater in rats fed *ad libitum* on the standard diet (0.55 gN) than in rats fed *ad libitum* on the MCT diet (0.46 gN) but this difference was not significant ($P>0.05$). When the daily intake of the standard diet was restricted to that of the tumour-bearing rats fed *ad libitum* on the MCT diet (Group B) the final host nitrogen content and tumour nitrogen content was similar to that of the tumour-bearing rats fed *ad libitum* on the MCT diet (Figure 3, Group C cf Group B). When non-tumour bearing rats were fed for 14 days on the MCT diet but restricted to a mean daily calorie intake of 9.1 Kcal. there was a slight, but not significant ($P>0.05$) decrease in the body nitrogen content (Figure 3, Group D).

**Figure 3** Body nitrogen content of rats before (hatched bars) and 14 days after tumour implantation (dotted bars). Final tumour nitrogen content is also shown (open bars). Animal groups were as defined in **Figure 2**. Values are mean ±s.e. ($n=6$).

*Blood glucose and ketone body concentrations of tumour-bearing rats on the various dietary regimes*

Table II shows the final blood glucose concentration and total ketone body concentration of rats fed *ad libitum* for 14 days on either the standard diet (Group A) or the MCT diet (Group B). A significant ketosis was observed in tumour-bearing rats fed *ad libitum* for 14 days on the MCT diet and this was accompanied by a significant ($P<0.02$) reduction in the blood glucose concentration. When the daily calorie intake of tumour-bearing rats fed on the standard diet was restricted to that observed in tumour-bearing rats fed *ad libitum* on the MCT diet the blood ketone body concentration was not affected but the blood glucose concentration was reduced to a level similar to that observed in tumour-bearing rats fed *ad libitum* on the MCT diet (Table II, Group C cf Group B). When the daily calorie intake of non-tumour bearing rats on the MCT diet was restricted to that of the tumour-bearing rats fed on the MCT diet the blood ketone concentration was similar but

**Table II** Total ketone body concentration (acetoacetate + 3-hydroxybutyrate) and glucose concentration in blood from rats 14 days after tumour implantation (Groups A, B and C) and in non-tumour-bearing rats (Group D)

| *Group* | *Total blood ketones (mM)* | *Blood glucose (mM)* |
|---|---|---|
| Control | 0.14±0.07 | 6.5±0.3 |
| A | 0.25±0.09 | 6.2±0.3 |
| B | 2.68±0.59 | 5.2±0.4 |
| C | 0.29±0.08 | 5.3±0.2 |
| D | 2.10±0.22 | 3.4±0.4 |

Control values refer to non-tumour-bearing rats fed *ad libitum* on the standard diet. Animal groups were as defined in **Figure 2**. Values are mean ± s.e. ($n=6$).

the blood glucose concentration was significantly reduced (Table II Group D cf Group B: $P<0.01$).

### Discussion

These results demonstrate clearly that induction of systemic ketosis following implantation of the Walker 256 carcinosarcoma in rats does not reduce the tumour growth rate, nor does it prevent the subsequent decrease in host body weight. Furthermore, sequential body composition analysis of the rats indicated that the host nitrogen content decreased to a similar extent in tumour-bearing animals fed on the MCT diet compared with those fed on the standard diet (Figure 3).

Two of the major enzymes responsible for the metabolism of ketone bodies in the mitochondria, 3-hydroxybutyrate dehydrogenase and acetyl CoA acetyltransferase, were shown to be present in significant amounts in the Walker 256 tumour (Table I). However, no activity of the enzyme 3-ketoacid CoA transferase was detected. A similar distribution of enzyme activities has been reported for other tumours of the peripheral tissues (Tisdale & Brennan, 1983). Since activity of the enzyme 3-ketoacid CoA transferase determines the extent to which 3-hydroxybutyrate is used as a metabolic fuel (Williamson *et al.*, 1971) it might be expected that the Walker 256 carcinosarcoma would be unable to metabolize ketone bodies. However, we have shown previously that Walker 256 tumour cells grown *in vitro* can form $^{14}CO_2$ from D(-)3-hydroxy (3-$^{14}$C-) butyrate, but only at a very low rate due to the presence of acetoacetyl CoA synthetase (Tisdale, 1984). Thus, although the tumour has a limited capacity for the metabolism of acetoacetate the low activity of acetoacetyl CoA synthetase and the absence of 3-ketoacid CoA transferase indicates that ketone bodies cannot be a major energy source for this tumour.

The diet chosen for use in these studies produced a significant systemic ketosis when fed to rats for only 3 days; this ketosis was maintained in rats fed on the MCT diet for at least 15 days (Figure 1). When non-tumour-bearing rats were fed on the MCT diet the increased concentration of ketone bodies in the blood was accompanied by a significant decrease ($P<0.01$) in the blood glucose concentration (Table II). This is consistent with the observation that ketosis induced in humans by a diet containing 85% of calories as fat is associated with a significant reduction in the blood glucose concentration (Phinney *et al.*, 1983). Similarly, the induction of a systemic ketosis by the infusion of 3-hydroxybutyrate into man (Sherwin *et al.*, 1975), dogs (Binkiewicz *et al.*, 1974) or sheep (Radcliffe *et al.*, 1983) has been shown to produce a significant decrease in the blood glucose concentration and is associated with a decrease in the rate of gluconeogenesis of both fed and fasted sheep (Radcliffe *et al.*, 1983). However, although a similar degree of ketosis was induced in both tumour bearing and non-tumour-bearing rats fed on the MCT diet (Figure 2) the decrease in the blood glucose concentration of tumour-bearing rats, though significant, was much less marked (Table II). Furthermore, the limited decrease in blood glucose concentration of tumour-bearing rats fed *ad libitum* on the MCT diet could be accounted for by the decrease in their daily calorie intake since tumour-bearing rats fed an equicaloric amount of the standard diet had a similar blood glucose concentration (Table II).

In septic sheep systemic ketosis is not accompanied either by a decrease in the blood glucose concentration or by a decrease in the rate of glucose production by the liver (Radcliffe *et al.*, 1983). This observation lends support to the suggestion that the metabolic abnormalities observed in the tumour-bearing host resemble some of those characteristic of semi-starvation but also some of those characteristic of sepsis and trauma (Brennan, 1977). The mechanism accounting for the failure of systemic ketosis to lower blood glucose in sepsis or cancer cachexia remains obscure. It may, however, be related to the insulin resistance characteristic of both metabolic states.

The failure of the ketogenic diet to restrict tumour growth may be, therefore, a result of the failure of ketosis to reduce the availability of glucose in tumour-bearing rats. It has already been shown that the growth rate of the Walker 256 tumour in rats can be decreased following administration of the glucose antimetabolite 2-deoxyglucose (Ball *et al.*, 1957) or following administration of hydrazine sulphate which inhibits gluconeogenesis (Gold, 1971). This suggests that the Walker 256 tumour is largely dependent upon glucose as an energy substrate. Thus, although the tumour cells are probably unable to use ketone bodies as a major energy source, the ketogenic diet did not restrict the supply of glucose to the tumour cells and thus failed to deprive the tumour of an important energy source.

The final tumour weight in rats fed *ad libitum* on the MCT diet was slightly, but not significantly ($P>0.05$) less than that in rats fed *ad libitum* on the standard diet (Figure 2, Group B cf Group A). The slight decrease in tumour growth rate can be accounted for entirely by the reduced calorie intake since a similar decrease in the final tumour size was observed in rats that had a daily allowance of the standard diet equal in calorie content to that eaten by rats fed on the MCT diet (Figure 2, Group C cf Group B).

There was a significant ($P<0.01$) decrease in the total nitrogen content of all tumour-bearing rats regardless of the dietary regimen (Figure 3). In all cases at least 60% of the host nitrogen loss could be accounted for by the nitrogen content of the tumour. This observation clearly supports the conclusion that the cachexia associated with the growth of the Walker 256 carcinosarcoma in rats is due mostly to the tumour acting as a "nitrogen trap" for the host's amino acids (Mider *et al.*, 1948).

Although we cannot conclude from this study that a host specific substrate does not restrict tumour growth, the results indicate the difficulty of such an approach to cancer treatment. The presence of metabolic abnormalities in cancer-bearing patients has been widely documented (see Fearon *et al.*, 1985) and these abnormalities alter the host's response to dietary modification. It is possible, however, that administration of a glucose antimetabolite or an inhibitor of gluconeogenesis as well as the ketogenic diet may be a means of providing a host specific substrate.

This work has been supported by a grant from the Cancer Research Campaign.

### References

BALL, H.A., WICK, A.N. & SANDERS, C. (1957). Influence of glucose antimetabolites on the Walker tumour. *Cancer Res.*, **17**, 235.

BERGMEYER, H.U. & BERNT, E. (1974). In: *Methods of Enzymatic Analysis Vol. 3*, p. 1205 (Ed. Bergmeyer). London and New York: Academic Press.

BINKIEWICZ, A., SADEGHI-NEJAD, A., HOCHMAN, H., LORIDAN, L. & SENIOR, B (1974). An effect of ketones on the concentrations of glucose and of free fatty acids in man independent of the release of insulin. *J. Paediatrics*, **84**, 226.

BRENNAN, M.F. (1977). Uncomplicated starvation versus cancer cachexia. *Cancer Res.*, **37**, 2359.

BRENNAN, M.F. (1981). Total parenteral nutrition in the cancer patient. *N. Engl. J. Med.*, **305**, 375.

CEDERBAUM, A.I. & RUBIN, E. (1976). Fatty acid excretion substrate shuttles and activities of the citric acid cycle in hepatocellular carcinomas of varying differentiation. *Cancer Res.*, **36**, 2980.

CONYERS, R.A.J., NEED, A.G., DURBRIDGE, T., HARVEY, N.D.M., POTENZY, N. & ROFFE, A.M. (1979). Cancer, ketosis and parenteral nutrition. *Med. J. Australia*, **1**, 398.

DEMETRAKOPOULOS, G.E. & BRENNAN, M.F. (1982). Tumoricidal potential of nutritional manipulations. *Cancer Res.*, **42**, (suppl.), 756s.

FEARON, K.C.H., PLUMB, J.A. & CALMAN, K.C. (1985). Nutritional consequences of cancer in man. *Clin. Nutr.*, (In Press).

GOLD, J. (1971). Inhibition of Walker 256 intramuscular carcinoma in rats by administration of hydrazine sulphate. *Oncology*, **25**, 66.

HOLROYDE, C.P., GABUZDA, T.G., PUTNAM, R.C., PAUL, P. & REICHARD, G.A. (1975). Altered glucose metabolism in metastatic carcinoma. *Cancer Res.*, **35**, 3710.

MAGEE, B.A., POTENZY, N., ROFE, A.M. & CONYERS, R.A.J. (1979). The inhibition of malignant cell growth by ketone bodies. *Aust. J. Exp. Biol. Med. Sci.*, **57**, 529.

MIDER, G.B., TESLUK, H. & MORTON, J.J. (1948). Effect of Walker Carcinoma 256 on food intake, body weight and nitrogen metabolism of growing rats, *Acta l'union Int. Contra Cancrum*, **6**, 409.

NIXON, D.W., HEYMSFIELD, S.B., COHEN, A.E. & 4 others. (1980). Protein calorie undernutrition in hospitalised cancer patients. *Am. J. Med.*, **68**, 683.

NIXON, D.W., LAWSON, D.H., KUTNER, M. & 6 others. (1981). Hyperalimentation of the cancer patient with protein-calorie undernutrition. *Cancer Res.*, **41**, 2038.

PEDERSEN, P.L. (1978). Tumour mitochondria and the biogenergetics of cancer cells. *Prog. Exp. Tumour Res.*, **22**, 190.

PHINNEY, S.D., BISTRIAN, B.R., WALFE, R.R. & BLACKBURN, G.L. (1983). The human metabolic response to chronic ketosis without calorie restriction: Physical and biochemical adaptation. *Metabolism*, **32**, 757.

PRESTON, T., REEDS, P.J., EAST, B.W. & HOLMES, P.H. (1985). A comparison of body protein determination in rats by *in vivo* neutron activation and carcass analysis. *Clin. Sci.*, **68**, 349.

RADCLIFFE, A.G., WILFE, R.R., COLPOYS, M.F., MUHLBACHER, F. & WILMORE, D.W. (1983). Ketone-glucose interaction in fed, fasted, and fasted-infected sheep. *Am. J. Physiol.*, **244**(s), R667.

ROBINSON, A.M. & WILLIAMSON, D.H. (1980). Physiological roles of ketone bodies as substrates and signals in mammalian tissue. *Physiol. Rev.*, **60**, 143.

SHERWIN, R.S., HENDLER, R.G. & FELIG, P. (1975). Effect of ketone infusions on amino acid and nitrogen metabolism in man. *J. Clin. Invest.*, **55**, 1382.

TISDALE, M.J. & BRENNAN, R.A. (1983). Loss of aceto-acetate coenzyme A transferase activity in tumours of peripheral tissues. *Br. J. Cancer*, **47**, 293.

TISDALE, M.J. (1984). Role of acetoacetyl-CoA synthetase in acetoacetate utilization by tumour cells. *Cancer Biochem. Biophys.*, **7**, 101.

WARREN, S. (1932). The immediate causes of death in cancer. *Am. J. Med. Sci.*, **184**, 610.

WATERHOUSE, C., JEANPRETRE, N. & KEILSON, J. (1979). Gluconeogenesis from alanine in patients with progressive malignant disease. *Cancer Res.*, **39**, 1968.

WILLIAMS, J.F. & MATTHAEI, K.I. (1981). Cancer induced body wasting. A review of cancer cachexia and a hypothesis concerning the molecular basis of the condition. *Asean J. Clin. Sci.*, **2**, 158.

WILLIAMSON, D.H., BATES, M.W., PAHE, M.A. & KREBS, H.A. (1971). Activities of enzymes involved in aceto-acetate utilization in adult mammalian tissues. *Biochem. J.*, **121**, 41.

WILLIAMSON, D.H. & MELLANBY, J. (1974). In: *Methods of Enzymatic Analysis* Vol. 4 p. 1836. Bergmeyer (Ed.) London: Academic Press.